# Associations of Autism Traits With Obsessive Compulsive Symptoms and Well-Being in Patients With Obsessive Compulsive Disorder: A Cross-Sectional Study

**DOI:** 10.3389/fpsyg.2021.697717

**Published:** 2021-07-30

**Authors:** Satomi Doi, Yuki Kobayashi, Yoshitake Takebayashi, Eriko Mizokawa, Atsuo Nakagawa, Masaru Mimura, Masaru Horikoshi

**Affiliations:** ^1^Department of Global Health Promotion, Tokyo Medical and Dental University, Tokyo, Japan; ^2^Japan Society for the Promotion of Science, Tokyo, Japan; ^3^Department of Neuropsychiatry, Keio University School of Medicine, Tokyo, Japan; ^4^Department of Health Risk Communication, School of Medicine, Fukushima Medical University, Fukushima, Japan; ^5^Nishimura Clinic, Tokyo, Japan; ^6^Clinical and Translational Research Center, Keio University Hospital, Tokyo, Japan; ^7^National Center for Cognitive Behavioral Therapy and Research, National Center of Neurology and Psychiatry, Tokyo, Japan

**Keywords:** autism traits, social skill, imagination, obsessive compulsive disorder, long-term, well-being

## Abstract

The aim of this study is to examine the association of autism traits with long-term obsessive compulsive disorder (OCD) symptoms and well-being levels in patient with OCD. Participants comprised 18 outpatients from a tertiary hospital and 100 adults who were registered in a large Japanese internet marketing research company and met OCD criteria by the Mini-International Neuropsychiatric Interview and were between the ages of 20 and 65 years. Clinical characteristics, autism trait assessed using the Autism Spectrum Quotient (AQ), OCD symptoms assessed using Yale-Brown Obsessive-Compulsive Scale (Y-BOCS), and well-being assessed using the Flourishing Scale were assessed. Multiple regression analyses showed that a greater total score of AQ, a greater subscale score “imagination” was associated with a greater score of Y-BOCS. Greater total score of AQ, a greater subscale score “social skill,” and “imagination” were associated with lower well-being score. Autism traits, especially lack of imagination, were associated with more severe OCD symptoms. Further, autism traits, especially social skill problems and lack of imagination, were associated with lower levels of well-being. Assessment of autism traits before treatment and a strategy designed for OCD patients with autism traits may be warranted.

## Introduction

### Obsessive-Compulsive Disorder

Obsessive-compulsive disorder (OCD) is a debilitating disorder and chronic condition (Steketee et al., 1999; Eisen et al., 2013). Effective short-term pharmacological and psychotherapeutic treatments for adult patients with OCD are well-established (Skapinakis et al., 2016; Guzick et al., 2018). However, a notable number of individuals experience residual OCD symptoms, which have been shown to follow the long-term rates of remission and relapse (Skoog and Skoog, 1999; Eisen et al., 2013; Kuehne et al., 2020).

### Long-Term Outcome of OCD

Though effective short-term OCD treatments have been established for adult patients, severe long-term OCD symptoms remain a major issue. For example, a meta-analysis of improvement and recovery rates recorded during long-term follow-ups ranging from 1 to 15 years revealed that about 50% of adults with OCD who received treatment including medication and cognitive behavioral therapy (CBT) did not show improvement or recovery (Sharma et al., 2014). Though the improvement and recovery rates varied according to the treatment (Sharma et al., 2014), advancements in treatments are required to decrease the rate of long-term OCD symptoms. In addition to improving long-term OCD symptoms, it is also important to focus on the positive aspects of a patient's status, such as subjective well-being which was defined as “cognitive evaluations or appraisals of life satisfaction as a whole, and emotional reaction to life events” by Diener and Diener (1995). Another study suggested that subjective well-being and psychological quality of life can be used interchangeably (Medvedev and Landhuis, 2018). Adults with OCD show impaired well-being compared to healthy controls (Subramaniam et al., 2013; Coluccia et al., 2016; Pozza et al., 2018), and the efficacy of CBT for well-being is not consistent (Subramaniam et al., 2013).

To improve the long-term OCD symptoms and well-being of adults with OCD, we must determine potential predictors of adverse prognoses. Possible predictors of long-term OCD symptoms include being male (Eisen et al., 2010, 2013), being unmarried (Steketee et al., 1999; Van Oudheusden et al., 2018), earlier age at onset and longer duration of untreated OCD (Eisen et al., 2013; Perris et al., 2021), longer duration of OCD (Eisen et al., 2013; Fineberg et al., 2013; Van Oudheusden et al., 2018), greater severity of symptoms (Eisen et al., 2013; Van Oudheusden et al., 2018), lower global functioning (Steketee et al., 1999; Nakajima et al., 2018), family accommodation (Nakajima et al., 2018), and hoarding symptoms (Nakajima et al., 2018).

### Autism Traits as Predictors of Long-Term OCD Outcomes

To consider the potential outcomes of OCD, the relevant transdiagnostic perspectives should be addressed. The majority of OCD patients have other psychiatric comorbidities such as other anxiety disorders, depression, and substance abuse (Gillan et al., 2017), which lead to lower adherence and response to treatment (Torres et al., 2016).

Previous studies have shown a high comorbidity of autism spectrum disorders (ASD) and OCD (Meier et al., 2015; Bedford et al., 2020) and an association between autism symptoms and OCD severity (Anholt et al., 2010; Arildskov et al., 2016). Specifically, a study by Meier et al. (2015), which followed 3,380,170 participants for 8 years, found that individuals with OCD were 13 times more likely to have comorbid ASD (6.6%) than those without OCD (0.5%). Further, OCD/ASD patients are less likely to respond to CBT (Murray et al., 2015; Tsuchiyagaito et al., 2017) and more likely to show lower remission rates (Mito et al., 2014; Murray et al., 2015; Tsuchiyagaito et al., 2017) even though previous studies with small sample sizes showed no differences in the severity of obsessions and compulsions between OCD patients and patients with OCD/ASD (Cath et al., 2008; Cadman et al., 2015; Wikramanayake et al., 2018). Therefore, autism traits may be key to predicting long-term OCD symptoms. Clinicians should pay attention to autism traits in adults with OCD to consider not only their response to treatment, but also their long-term symptoms. This study focused on autism traits, rather than a diagnosis of ASD, because the genetic and biological etiology among ASD patients and the general population overlap (Bralten et al., 2018). Clinically, however, it is helpful to focus on both a diagnosis of ASD and autism traits in OCD patients.

Autism traits include five dimensions: lack of social skills, attention switching problems, attention to detail, communication problems, and lack of imagination. Notably, attention switching problems (e.g., getting too strongly absorbed in one task) may be associated with long-term OCD symptoms and decreased well-being. The ability to flexibly switch between stimuli, which is related to cognitive inflexibility (Worringer et al., 2019), is evaluated by using task switching or set-shifting. During task performance, individuals with ASD show altered brain activation and dynamics compared to non-ASD individuals (Uddin, 2021). Furthermore, attention switching is related to executive function (EF). EF includes several areas such as inhibition, attention, working memory, flexibility, planning, monitoring, preparatory processing, fluency, and formation (Miyake et al., 2000; Craig et al., 2016). Individuals with ASD are more likely to show impairment of attention compared to children without ASD (Craig et al., 2016). Anholt et al. (2010) showed that attention switching problems predict OCD symptoms in adults with OCD. Further, it has been reported that patients with OCD/ASD had lower attention switching skills than those with only OCD. Additionally, both patients with OCD/ASD and those with OCD alone showed lower attention switching skills than healthy controls (Cath et al., 2008). These previous findings were not limited to long-term symptoms. It may be helpful to explore which dimension of autism traits relates to long-term OCD symptoms and well-being to develop an appropriate treatment.

### Aim and Hypotheses

We aimed to examine the association of autism traits with OCD symptoms and well-being in adults with OCD, who had started CBT treatment more than 1 year prior. We defined the following four hypotheses:

1) Autism traits are associated with higher OCD symptoms.
2) Autism traits are associated with lower levels of well-being.
3) Among the autism traits, problems with attention switching are associated with higher OCD symptoms.
4) Among the autism traits, problems with attention switching are associated with lower levels of well-being.

## Materials and Methods

### Participants

The data were collected as part of the Program for OCD Prognosis Improvement (POP-I) study, a cross-sectional study. Participants were recruited in two ways from November 2017 to June 2018. Inclusion criteria were: participants (1) who had a primary diagnosis of OCD by a psychiatrist or met the OCD criteria put forth by the Mini-International Neuropsychiatric Interview and (2) who started any treatment for OCD more than 1 year prior. Exclusion criteria were: participants (1) who started any treatment for OCD within 1 year and (2) who had missing values for the time of treatment initiation. We defined long-term OCD symptoms as being present for 1 year or longer, based on a previous study (Sharma et al., 2014).

**Figure 1:**
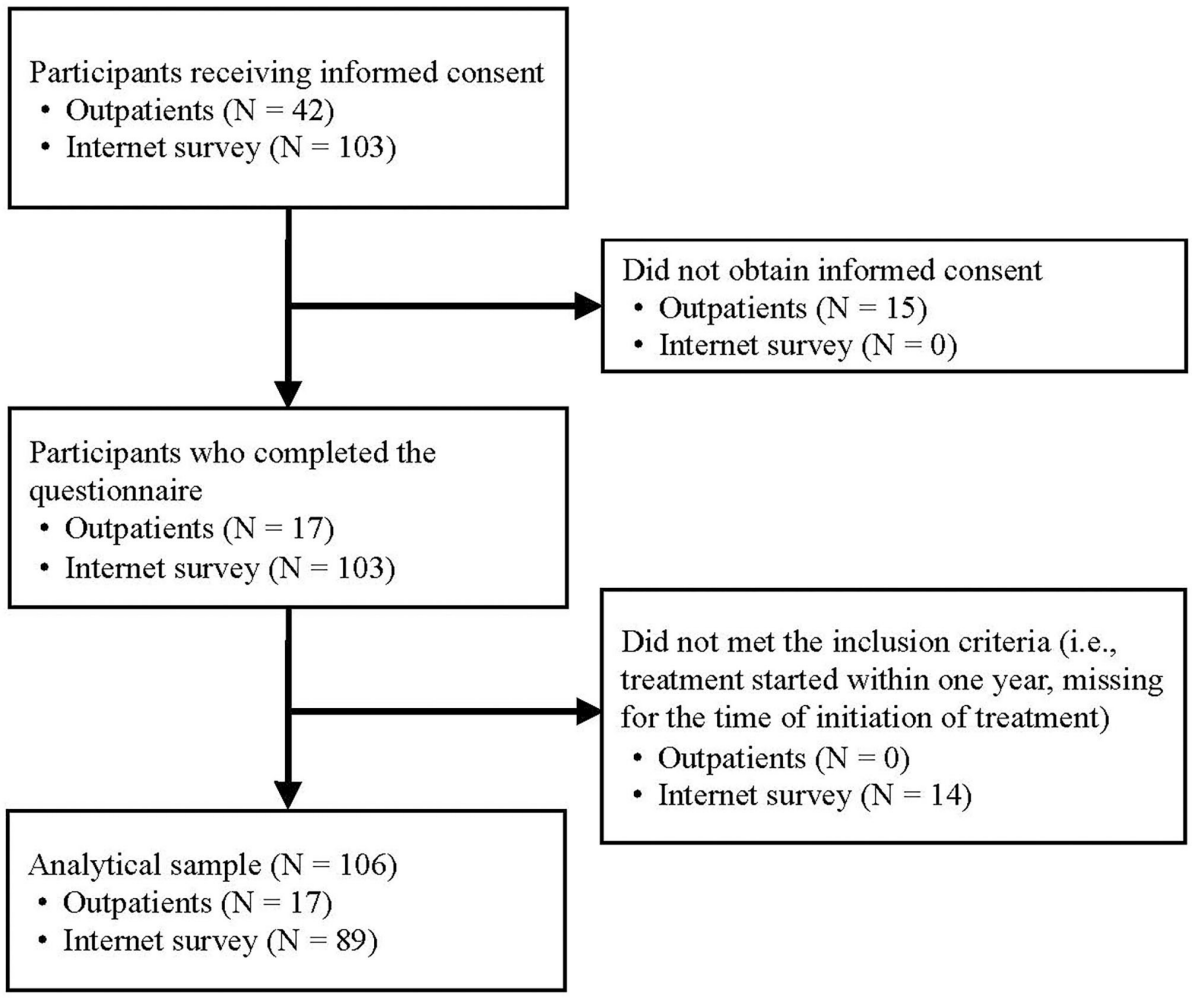
Requirement flow chart.

First, we orally requested informed consent from 42 individuals with OCD who were outpatients at the National Center Hospital in Tokyo, Japan, and who had completed a manual CBT program for OCD, consisting of 12 sessions of exposure and response prevention (Shinmei et al., 2017) and 16 sessions of family-based exposure and response prevention (Kobayashi et al., 2020) at the National Center Hospital more than 1 year prior. We obtained written informed consent from 17 of the 42 (response rate = 40.5%) and distributed the questionnaire via mail. Second, we recruited participants through an internet survey of 103 individuals registered with a large Japanese internet marketing research company who met the OCD criteria set forth by the Mini-International Neuropsychiatric Interview and who had started treatment for OCD more than 1 year prior; all 103 individuals completed the questionnaire online. Information on the study, including background, purpose, and policies for the management of personal data, was immediately provided to participants. Those who answered all questions were deemed to have provided informed consent (Figure 1).

A posteriori power analysis was conducted. The minimum sample size for the multiple regression model was suggested as either 25 or more based on Jenkins and Quintana-Ascencio's simulation (Jenkins and Quintana-Ascencio, 2020), or 24 based on the two subject per variable (SPV) criterion (total number of explanatory variables in the regression model in this study = 12*2) (Austin and Steyerberg, 2015). Thus, we collected more data than this minimum sample size, from the maximum number of people possible with our budget. Therefore, 120 OCD patients were enrolled in this study. However, we excluded 14 of these participants because they did not meet our inclusion criteria, due to either starting their treatments within <1 year or having missing data for the time of treatment initiation, so 106 participants were included in the final analysis. We calculated the required minimum sample size with power = 0.90 and alpha = 0.05 using G*power software (University of Düsseldorf, Düsseldorf, Germany) (Faul et al., 2007) based on Cohen's formula (Cohen, 1988). A significant effect size (*f*^2^ = 0.25) in a multiple regression model with 12 indicators, required a sample size of 99, which our sample size exceeds.

### Measurements

#### Autism Traits

Autism traits were assessed using the Japanese version of the autism spectrum quotient (AQ) (Wakabayashi et al., 2004), which is a self-reported screening instrument developed by Baron-Cohen et al. (2001) that has five domains (social skills, attention switching, attention to detail, communication, and imagination). Participants were asked to rate 50 items on a scale of 1 (*definitely agree*) to 4 (*definitely disagree*). A higher score indicated more severe autism traits. The Cronbach's alpha for the Japanese version of the AQ is 0.81, indicating good internal consistency (Wakabayashi et al., 2004). The Cronbach's alpha for the AQ was 0.88.

#### OCD Symptoms

The severity of obsessions and compulsions was assessed using the Japanese version of the Self-Report Yale-Brown Obsessive-Compulsive Scale (Y-BOCS) (Hamagaki et al., 1999), which is translated form the English version (Baer, 1991). This scale consists of 10 items graded on a scale of 0 (no symptoms) to 4 (extremely severe symptoms). The Cronbach's alpha for the Japanese version of the Self-Report Y-BOCS is 0.90, indicating good internal consistency (Hamagaki et al., 1999). The Cronbach's alpha for the Y-BOCS was 0.89.

#### Well-Being

Well-being was assessed using 10 items: emotional stability, vitality, resilience, optimism, positive emotions, self-esteem, engagement, meaning, positive relationships, and competence (Murakami et al., 2020). Positive emotion was rated according to a scale ranging from 0 (*extremely unhappy*) to 10 (*extremely happy*). Emotional stability and vitality were rated according to a scale ranging from 0 (*none*) to 3 (*all the time*). The other seven items were rated according to a scale ranging from 0 (*strongly disagree*) to 4 (*strongly agree*). This scale is a self-reported questionnaire and has been shown to have good reliability (Murakami et al., 2020). The Cronbach's alpha was 0.80.

#### Covariates

The questionnaire assessed sex (“male” or “female”), age, marital status (“unmarried,” “married,” “divorced,” “separated, or “other”), living situation (“living with family,” “living with someone other than family” or “other”), household income (“ <2,000,000 yen,” “2,000,000- <4,000,000 yen,” “4,000,000- <6,000,000 yen,” “6,000,000- <8,000,000 yen,” “8,000,000- <10,000,000 yen,” “10,000,000+ yen,” or “unknown”; 1 million yen ~ USD 10,000), education level (“junior high school,” “high school,” “vocational college,” “junior college,” “college,” “graduate school,” “in school,” or “other”), job status (“full-time,” “part-time,” “leave of absence,” “not working,” “housewife,” or “other”), and duration of untreated illness (days).

### Ethics

This study was approved by the ethics committee at the National Center of Neurology and Psychiatry in Tokyo, Japan (No. A2017-064).

### Statistics

First, we compared the different demographic characteristics between the outpatients and internet-recruited participants using the Chi-squared and *t*-tests. Second, Pearson's correlation was used to examine the correlations between total AQ score and each subscale, Y-BOCS total, and well-being total score. Third, we conducted multiple regression analyses to explore the association between autism traits and OCD symptoms. Analyses were conducted for total AQ score and each subscale score (i.e., social skills, attention switching, attention to detail, communication, and imagination). After estimating simple linear regression analyses (crude model), multiple linear regression analyses were performed, adjusting for covariates including sex, age, marital status, living situation, household income, education level, job status, duration of untreated illness, and recruiting method (Model 1). We also adjusted for the well-being score, which is a possible mediator (Model 2). Fourth, we conducted multiple regression analyses to explore the association between autism traits and well-being levels. In Model 2, we adjusted for OCD symptoms. Fifth, we conducted multiple linear regressions using only the participants who were recruited through the internet-survey as sensitivity analysis. Variance Inflation Factor (VIF) values were calculated to assess the multicollinearity for regression models, where VIF>10 was considered problematic (Vittinghoff et al., 2012). In the analyses, missing data were treated as dummy variables (i.e., we converted a missing value into a numerical value such as “99”). All analyses were conducted using STATA version 15.0 SE.

## Results

### Participants' Characteristics

Supplementary Table 1 shows the participants' characteristics; 37 participants (34.9%) were male (female: *N* = 69, 65.1%), and the mean age was 39.95 ± 10.19 years (from 20 to 78 years old). The mean age was 42.78 ± 10.37 years for males and 38.43 ± 9.84 years for females. The Chi-squared test showed that participants who were outpatients were more likely to report their household income as “unknown” than those who were recruited through the internet-survey (*p* = 0.01). Other characteristics were not significantly different. Additionally, AQ total score, AQ sub-scale scores, Y-BOCS total score, and well-being total score are shown in Supplementary Table 1. We also show the distribution of the AQ, Y-BOCS, and well-being total scores (Supplementary Figure 1). All scores, except the subscale scores for local details were higher, and well-being score was lower in the internet-survey participants compared to the outpatients.

### Correlations

Table 1 shows the score ranges and correlations between AQ total score, AQ subscale scores (i.e., social skill, attention switching, attention to detail, communication, and imagination), Y-BOCS total score, and well-being total score. Social skills, attention switching, communication, and imagination were positively correlated with each other. In contrast, attention to detail was not significantly correlated with social skills, and communication was weakly correlated with attention switching and imagination. Y-BOCS total score was positively correlated with AQ total score, social skill, attention switching, communication, and imagination. Well-being total score was negatively correlated with AQ total score, social skill, attention switching, communication, imagination, and Y-BOCS total score. However, attention to detail was not associated with either Y-BOCS and well-being total scores.

**Table 1:** The results of Pearson's correlation.

	**Range of score**		**1**		**2**		**3**		**4**		**5**		**6**		**7**	
	**Min**	**Max**	**r**	***p***	**r**	***p***	**r**	***p***	**r**	***p***	**r**	***p***	**r**	***p***	**r**	***p***
1. AQ total score	0	50	1.00													
2. Social skill	0	10	0.75	<0.001	1.00											
3. Attention switching	0	10	0.77	<0.001	0.40	<0.001	1.00									
4. Attention to detail	0	10	0.16	0.110	−0.18	0.061	0.19	0.048	1.00							
5. Communication	0	10	0.84	<0.001	0.57	<0.001	0.54	<0.001	−0.04	0.666	1.00					
6. Imagination	0	10	0.61	<0.001	0.46	<0.001	0.35	<0.001	−0.38	<0.001	0.51	<0.001	1.00			
7. Y-BOCS total score	0	40	0.46	<0.001	0.29	0.003	0.42	<0.001	0.11	0.253	0.36	<0.001	0.29	0.003	1.00	
8. Well-being total score	0	44	−0.43	<0.001	−0.43	<0.001	−0.27	0.005	0.03	0.764	−0.35	<0.001	−0.29	0.003	−0.25	0.009

*AQ, Autism-Spectrum Quotient; Y-BOCS, Yale-Brown obsessive compulsive scale*.

### Association Between Autism Traits and OCD Symptoms

Table 2 shows the results of linear regression analyses to examine whether AQ total score and each AQ subscale score are associated with Y-BOCS total score. In the crude model, total AQ score and all subscale scores except “attention to detail” were significantly associated with Y-BOCS score. In Model 1, which adjusted for confounders, a higher total AQ score (β = 0.37, 95% confidence interval [CI] = 0.11–0.62) and higher subscale score for “imagination” (β = 0.97, 95% CI = 0.06–1.89) were positively associated with OCD symptoms. The coefficients of the associations between total AQ score with Y-BOCS remained, even after adjusting for the well-being score (Model 2).

**Table 2:** Association between AQ Total score and each sub-scale score and Y-BOCS.

**Predictors**	**Crude model**	**Model 1^a^**	**Model 2^b^**
	**β (95% CI)**	**β (95% CI)**	**β (95% CI)**
AQ Total score	**0.53 (0.33–0.73)**	**0.37 (0.11–0.62)**	**0.36 (0.09–0.62)**
	Adjusted *R*^2^ = 0.21	Adjusted *R*^2^ = 0.23	Adjusted *R*^2^ = 0.22
AQ subscale: Social skill	**0.93 (0.33–1.53)**	0.63 (−0.04 to 1.30)	0.58 (−0.13 to 1.29)
	Adjusted *R*^2^ = 0.07	Adjusted *R*^2^ = 0.19	Adjusted *R*^2^ = 0.18
AQ subscale: Attention switching	**1.76 (1.03–2.50)**	0.91 (−0.02 to 1.84)	0.88 (−0.06 to 1.81)
	Adjusted *R*^2^ = 0.17	Adjusted *R*^2^ = 0.19	Adjusted *R*^2^ = 0.19
AQ subscale: Attention to detail	0.44 (−0.32 to 1.21)	0.19 (−0.65 to 1.02)	0.20 (−0.64 to 1.04)
	Adjusted *R*^2^ = 0.003	Adjusted *R*^2^ = 0.13	Adjusted *R*^2^ = 0.13
AQ subscale: Communication	**1.13 (0.57–1.70)**	0.66 (−0.01 to 1.33)	0.62 (−0.07 to 1.30)
	Adjusted *R*^2^ = 0.12	Adjusted *R*^2^ = 0.19	Adjusted *R*^2^ = 0.18
AQ subscale: Imagination	**1.19 (0.42–1.96)**	**0.97 (0.06–1.89)**	0.91 (−0.03 to 1.85)
	Adjusted *R*^2^ = 0.07	Adjusted *R*^2^ = 0.19	Adjusted *R*^2^ = 0.19

*Y-BOCS, Yale-Brown obsessive compulsive scale; AQ, Autism-spectrum quotient; FAS-SR, Family accommodation scale for OCD-self rated version*.

a *Model 1 added sex, age, marital status, living with someone, household income, educational level, job status, time between appearance of symptoms and visiting hospital, FAS-PR score, and recruiting method into crude model*.

b *Model 2 added well-being score into Model 1. The bold values indicate p < 0.001*.

In the sensitivity analyses (Supplementary Table 2), the internet-survey participants showed a similar coefficient of total AQ score (Model 2: β = 0.38, 95% CI = 0.08–0.68). Contrastingly, subscale score for “communication” was positively associated with Y-BOCS instead of “imagination” (Model 2: β = 0.78, 95% CI = 0.03–1.53).

### Association Between Autism Traits and Well-Being

Table 3 shows the results of linear regression analyses to examine whether AQ total score and each AQ subscale score are associated with well-being total score. The mean well-being score was 13.1 (SD = 6.1). The crude model also showed that the total AQ score and all subscale scores except for “attention to detail” were negatively associated with well-being. In Model 2, a higher total AQ score (β= −0.23, 95% CI = −0.42 to −0.04), a higher subscale score for “social skills” (β = −0.72, 95% CI = −1.20 to −0.24), and a higher subscale score for “imagination” (β = −0.71, 95% CI = −1.38 to −0.03) were negatively associated with well-being. The coefficients of the total AQ score and “social skills” score remained, even after adjusting for the Y-BOCS score.

**Table 3:** Association between AQ Total score and each sub-scale score and well-being.

**Predictors**	**Crude model**	**Model 1^a^**	**Model 2^b^**
	**β (95% CI)**	**β (95% CI)**	**β (95% CI)**
AQ Total score	**−0.36 (−0.51 to −0.21)**	**−0.23 (−0.42 to −0.04)**	**−0.22 (−0.43 to −0.02)**
	Adjusted *R*^2^ = 0.17	Adjusted *R*^2^ = 0.21	Adjusted *R*^2^ = 0.21
AQ subscale: Social skill	**−1.03 (−1.45 to −0.62)**	**−0.72 (−1.20 to −0.24)**	**−0.70 (−1.19 to −0.21)**
	Adjusted *R*^2^ = 0.18	Adjusted *R*^2^ = 0.24	Adjusted *R*^2^ = 0.23
AQ subscale: Attention switching	**−0.83 (−1.41 to −0.25)**	−0.26 (−0.95 to 0.44)	−0.19 (−0.90 to 0.53)
	Adjusted *R*^2^ = 0.06	Adjusted *R*^2^ = 0.16	Adjusted *R*^2^ = 0.16
AQ subscale: Local details	0.09 (−0.48 to 0.66)	0.11 (−0.50 to 0.71)	0.12 (−0.48 to 0.73)
	Adjusted *R*^2^ = −0.01	Adjusted *R*^2^ = 0.16	Adjusted *R*^2^ = 0.16
AQ subscale: Communication	**−0.81 (−1.23 to −0.38)**	−0.37 (−0.87 to 0.14)	−0.33 (−0.84 to 0.19)
	Adjusted *R*^2^ = 0.11	Adjusted *R*^2^ = 0.18	Adjusted *R*^2^ = 0.17
AQ subscale: Imagination	**−0.89 (−1.46 to −0.32)**	**−0.71 (−1.38 to −0.03)**	−0.66 (−1.36 to 0.04)
	Adjusted *R*^2^ = 0.08	Adjusted *R*^2^ = 0.20	Adjusted *R*^2^ = 0.19

*Y-BOCS, Yale-Brown obsessive compulsive scale; AQ, Autism-spectrum quotient; FAS-SR, Family accommodation scale for OCD-self rated version*.

a *Model 1 added sex, age, marital status, living with someone, household income, educational level, job status, time between appearance of symptoms and visiting hospital, FAS-PR score, and recruiting method into crude model*.

b *Model 2 added Y-BOCS score into Model 1. The bold values indicate p < 0.001*.

In the sensitivity analyses (Supplementary Table 3), internet-recruited participants showed no association between total AQ score and well-being after adjusting for covariates. The subscale scores for “social skills” and “imagination” showed negative association after adjusting for covariates (social skills: β = −0.66, 95% CI = −1.25 to −0.07; imagination: −0.79, 95% CI = −1.57 to −0.01). The range of VIF values for each indicator in the regression models was less than four.

## Discussion

### Summary of Results

We found that autism traits were associated with OCD symptoms and lower levels of well-being among OCD patients. Particularly, lack of imagination was associated with OCD symptoms, and lower social skills and lack of imagination were associated with lower levels of well-being, even after adjusting for participant characteristics and the duration of untreated illness. This indicates that autism traits may be a notable predictive factor for long-term symptoms, independent of other major factors.

Our results showed that autism traits were associated with OCD symptoms and lower levels of well-being, consistent with our first two hypotheses. Previous studies have found that OCD/ASD patients are more likely to show lower remission rates (Mito et al., 2014; Murray et al., 2015; Tsuchiyagaito et al., 2017). Additionally, our findings indicate that OCD patients with higher levels of autism traits, irrespective of ASD diagnosis, may be more likely to show long-term symptoms and decreased well-being.

However, our results do not support our 3rd and 4th hypotheses regarding the association between attention switching problems with higher OCD symptoms and lower well-being, contradicting previous studies that suggested that attention switching problems are a key factor to related to OCD symptoms (Cath et al., 2008; Anholt et al., 2010). However, some studies showed that OCD patients' cognitive flexibility was improved by treatment (Vandborg et al., 2012; Jalal et al., 2018). Thus, our results indicate that OCD treatment, including CBT, might diminish the association between attention switching problems and symptoms. Thus, further studies with a larger sample size which account for the different treatment types (e.g., CBT, CBT + medication, only medication, or other non-drug treatments), are needed.

Among the five dimensions of autism traits, we found that a lack of imagination was associated with higher OCD symptoms. A lack of imagination could reflect a lack of self-insight, which is related to a deficit in theory of mind. Patients with OCD showed a deficit in theory of mind and decreased ability to regulate emotions (Yazici and Yazici, 2019). Particularly, OCD patients with poor insight were more likely to have difficulty with emotional regulation, which may induce a higher level of OCD symptoms. Lack of imagination was also associated with lower well-being.

Additionally, we found that patients with lower social skills were more likely to report lower levels of well-being, even after controlling for OCD symptoms. To our knowledge, no previous study has examined the association between autism traits and long-term well-being among OCD patients. Social skills are a major predictive factor related to psychological well-being and quality of life (Segrin and Taylor, 2007; Segrin et al., 2007; Muller et al., 2015). We found a moderate association between social skills and imagination, both of which may be important for social interactions. This is supported by a study by Tobin et al. (2014), which reported that in patients with ASD, higher social skills and imagination facilitated social functioning and participation, resulting in improved well-being and quality of life.

Additionally, we conducted the sensitivity analyses using only internet-survey participants and found similar results, i.e., lower social skills and lack of imagination were associated with lower well-being. However, lower level of communication was only associated with OCD symptoms. The subscale score for “communication” was moderately correlated with “social skills,” indicating that patients who have more social participation may be more aware of their social difficulties, which lead to a higher score for “communication.” Although we could not assess the participants' social connection, the internet-survey participants might interact with others and struggle with communication with others. To clarify which autism traits predict long-term OCD symptoms and well-being, further studies with larger sample sizes are needed.

### Implications

This is the first study to find that a lack of imagination was associated with long-term OCD symptoms and that lower social skills and lack of imagination were associated with lower levels of well-being among adult patients with OCD who had been treated for OCD for at least 1 year. Our findings indicate that it may be helpful to evaluate autism traits not only before but also after OCD treatment to estimate the patient's prognosis. Furthermore, these findings may aid in the development of a new treatment to ensure a better patient prognosis. Typically, CBT for OCD includes ERP (Olatunji et al., 2013), identification and modification of dysfunctional beliefs that impact OCD symptoms (Mckay et al., 2015), and improvement of family accommodations (Thompson-Hollands et al., 2014). However, for OCD patients with autism traits, this may be insufficient. In addition to the usual CBT for OCD, such patients may benefit from interventions to promote social skills and imagination. Social skills training, which was developed for ASD patients (Gantman et al., 2012), may be useful to improve social skills and can be delivered in various ways: as group-based training (Laugeson et al., 2014; Gates et al., 2017), an online self-administered training (Lehenbauer et al., 2013), or caregiver-assistant training (Laugeson et al., 2015). Further, well-being therapy (Fava, 1999; Ruini and Fava, 2009) may be used to improve well-being levels among OCD patients with autism traits. A drama-based intervention designed to promote imagination in children by participating in fictional frameworks may be helpful to encourage social communication and imaginative behaviors (Beadle-Brown et al., 2018). Further studies are needed to determine the best way to incorporate interventions to promote social skills and imagination into CBT for OCD patients with autism traits. For example, after normal CBT for all OCD patients, the clinician provides online self-administered social skill training programs for those with autism traits. Clinicians can also encourage OCD patients with autism traits to engage in social activities, perhaps in the workplace.

### Limitations

This study had several limitations. First, the sample may have been biased, as we recruited patients from one hospital and used data from an internet survey. Even though multiple linear regression models adjusted for the difference of recruiting methods and sensitivity analyses were performed, sampling bias is still a major limitation of this study. Further, the response rate of participants recruited at the hospital was low (40.5%). Thus, patients with a better prognosis may have been more likely to participate. Second, although we only included participants who met OCD criteria of the MINI, we could not confirm that internet-survey participants had been diagnosed with OCD by a psychiatrist. Further studies, where the psychiatrists confirm the patient's diagnosis, are needed. Third, we did not measure confounders such as depression and anxiety, which are highly comorbid with OCD (Gillan et al., 2017). Severe depression and anxiety might mimic the effect of autism traits and long-term OCD symptoms. Additionally, other confounders may include the presence of intellectual disability of symptomatic epilepsy origin; parents' ASD diagnosis and age (Modabbernia et al., 2017; Strasser et al., 2018); and social connection. Fourth, there is an information bias, as all measurements in this study were self-reported. Although the Cronbach's alpha of the self-report version of the Y-BOCS in this study was good, there are several inconsistencies between the self-report and clinician-administered version of the Y-BOCS (Federici et al., 2010). Additionally, attention switching can be assessed using task switching or set-shifting, which can evaluate the ability of attention switching objectively. Thus, our findings should be interpreted carefully. Further studies using objective assessments of autism traits and OCD symptoms are needed.

### Conclusions

OCD patients with more autism traits, especially those who lack imagination, were more likely to show severe long-term OCD symptoms. Further, the OCD patients with more autism traits, especially who have problem with social skills and a lack of imagination, were more likely to show lower levels of well-being. Assessment of autism traits before OCD treatment and a strategy designed for OCD patients with autism traits may be warranted.

## Data Availability Statement

The datasets are available by request to the corresponding author and approving by the ethics committee. Requests to access the datasets should be directed to ykobayashi@keio.jp.

## Ethics Statement

The studies involving human participants were reviewed and approved by National Center of Neurology and Psychiatry. The patients/participants provided their written informed consent to participate in this study.

## Author Contributions

YK, YT, and MH designed the study. YK and EM managed administration of the study, including the ethical review process. SD and YT analyzed data. SD drafted the manuscript. YK, YT, EM, AN, MM, and MH provided critical comments on the manuscript related to intellectual content. All authors have read and approved the final manuscript.

## Conflict of Interest

The authors declare that the research was conducted in the absence of any commercial or financial relationships that could be construed as a potential conflict of interest.

## Publisher's Note

All claims expressed in this article are solely those of the authors and do not necessarily represent those of their affiliated organizations, or those of the publisher, the editors and the reviewers. Any product that may be evaluated in this article, or claim that may be made by its manufacturer, is not guaranteed or endorsed by the publisher.
