# Supplementary material for: Associations of Autism Traits With Obsessive Compulsive Symptoms and Well-Being in Patients With Obsessive Compulsive Disorder: A Cross-Sectional Study
Source: Front Psychol. 2021 Jul 30;12:697717. doi: 10.3389/fpsyg.2021.697717 (PMC8360877; doi:10.3389/fpsyg.2021.697717)
Supplement: Supplementary file 1 [file Data_Sheet_1.docx]

| Supplementary Table 1 Demographic characteristics | |  |  |  |  |  |  |  |
| --- | --- | --- | --- | --- | --- | --- | --- | --- |
|  |  | Total (N = 106) | | Outpatients | | Internet-survey | | p for Chi-squared test or t-test |
|  |  |  |  | Total (N = 17) | | Total (N = 89) | |  |
|  |  | N | % or mean (SD) | N | % or mean (SD) | N | % or mean (SD) |  |
| Sex | Male | 37 | 34.9 | 4 | 23.5 | 33 | 37.1 | 0.283 |
|  | Female | 69 | 65.1 | 13 | 76.0 | 56 | 62.9 |  |
| Age |  | 106 | 39.95 (10.19) | 17 | 38.12 (12.25) | 89 | 40.30 (9.79) | 0.420 |
| Marital status | Unmarried | 56 | 52.8 | 9 | 52.9 | 47 | 52.8 | 0.213 |
|  | Married | 37 | 34.9 | 6 | 35.3 | 31 | 34.8 |  |
|  | Divorce | 11 | 10.4 | 1 | 5.9 | 10 | 11.2 |  |
|  | Separation | 1 | 0.9 | 0 | 0 | 1 | 1.1 |  |
|  | Other | 1 | 0.9 | 1 | 5.9 | 0 | 0 |  |
| Living with someone | No | 18 | 17.0 | 1 | 5.9 | 17 | 19.1 | 0.183 |
|  | Yes | 88 | 83.0 | 16 | 94.1 | 72 | 80.9 |  |
| Household income (yen)* | <2 million | 13 | 12.3 | 0 | 0 | 13 | 14.6 | 0.010 |
|  | 2 million-<4 million | 26 | 24.5 | 3 | 17.6 | 23 | 25.8 |  |
|  | 4 million-<6 million | 17 | 16.0 | 2 | 11.8 | 15 | 16.8 |  |
|  | 6 million-<8 million | 11 | 10.4 | 2 | 11.8 | 9 | 10.1 |  |
|  | 8 million+ | 15 | 14.2 | 2 | 11.8 | 13 | 14.6 |  |
|  | Unknown | 17 | 16.0 | 8 | 47.1 | 9 | 7.9 |  |
|  | Missing | 7 | 6.6 | 0 | 0 | 7 | 6.6 |  |
| Educational level | High school or less | 42 | 39.5 | 6 | 35.3 | 36 | 40.4 | 0.160 |
|  | Some college | 12 | 11.3 | 2 | 11.8 | 10 | 11.2 |  |
|  | College or more | 45 | 42.4 | 8 | 47.1 | 37 | 41.6 |  |
|  | Attending a school | 6 | 5.7 | 0 | 0 | 6 | 6.7 |  |
|  | Other/Unknown | 1 | 0.9 | 1 | 5.9 | 0 | 0 |  |
| Job status | Full-time | 27 | 25.5 | 3 | 17.6 | 24 | 25.0 | 0.661 |
|  | Part-time | 11 | 10.4 | 2 | 11.8 | 9 | 10.1 |  |
|  | Not working | 56 | 52.8 | 11 | 64.7 | 45 | 50.6 |  |
|  | Other | 12 | 11.3 | 1 | 5.9 | 11 | 12.4 |  |
| Time between appearance of symptoms and visiting hospital (days) |  | 106 | 113.47 (120.46) | 17 | 82.00 (124.54) | 89 | 119.48 (119.44) | 0.242 |
| FAS-SR total score |  | 106 | 11.04 (12.76) | 17 | 11.00 (12.39) | 89 | 11.04 (12.39) | 0.990 |
| AQ total score |  | 106 | 29.06 (7.21) | 17 | 22.65 (8.14) | 89 | 30.8 (6.36) | <0.001 |
| AQ subscale: Social skill |  | 106 | 6.19 (2.56) | 17 | 4.53 (2.94) | 89 | 6.51 (2.37) | 0.003 |
| AQ subscale: Attention switching |  | 106 | 6.75 (1.98) | 17 | 5.35 (2.32) | 89 | 7.02 (1.80) | 0.001 |
| AQ subscale: Local details |  | 106 | 5.93 (2.09) | 17 | 5.65 (2.34) | 89 | 5.99 (2.04) | 0.538 |
| AQ subscale: Communication |  | 106 | 5.51 (2.63) | 17 | 3.24 (2.39) | 89 | 5.94 (5.43) | <0.001 |
| AQ subscale: Imagination |  | 106 | 4.67 (1.99) | 17 | 3.88 (1.76) | 89 | 4.82 (2.01) | 0.075 |
| Y-BOCS total score |  | 106 | 19.93 (8.27) | 17 | 16.29 (7.77) | 89 | 20.63 (8.22) | 0.047 |
| Well-being total score |  | 106 | 13.07 (6.12) | 17 | 18.35 (4.53) | 89 | 12.06 (5.87) | <0.001 |
| Note. SD=Standard deviation, FAS-SR=Family accommodation scale for OCD-self rated version, AQ=Autism-Spectrum Quotient, Y-BOCS=Yale-Brown obsessive compulsive scale. | | | | | | | | |
|  |  |  |  |  |  |  |  |  |
| *1 million yen is equivalent to USD 10,000. | |  |  |  |  |  |  |  |

| Supplementary Table 2 Association between AQ Total score and each sub-scale score and Y-BOCS using internet-survey participants. | | | |
| --- | --- | --- | --- |
| Predictors | Crude model | Model 1^a^ | Model 2^b^ |
|  | *β* (95%CI) | *β* (95%CI) | *β* (95%CI) |
| AQ Total score | **0.53 (0.28 to 0.78)** | **0.39 (0.10 to 0.68)** | **0.38 (0.08 to 0.68)** |
|  | Adjusted R^2^ = 0.16 | Adjusted R^2^ = 0.18 | Adjusted R^2^ = 0.17 |
| AQ subscale: Social skill | **0.78 (0.06 to 1.50)** | 0.63 (−0.16to 1.41) | 0.58 (−0.24 to 1.40) |
|  | Adjusted R^2^ = 0.04 | Adjusted R^2^ = 0.12 | Adjusted R^2^ = 0.11 |
| AQ subscale: Attention switching | **1.56 (0.65 to 2.47)** | 0.74 (−0.31 to 1.79) | 0.73 (−0.33 to 1.78) |
|  | Adjusted R^2^ = 0.11 | Adjusted R^2^ = 0.11 | Adjusted R^2^ = 0.11 |
| AQ subscale: Attention to detail | 0.41 (−0.44 to 1.27) | 0.25 (−0.64 to 1.15) | 0.27 (−0.63 to 1.17) |
|  | Adjusted R^2^ = –0.001 | Adjusted R^2^ = 0.09 | Adjusted R^2^ = 0.09 |
| AQ subscale: Communication | **1.07 (0.40 to 1.74)** | **0.81 (0.07 to 1.55)** | **0.78 (0.03 to 1.53)** |
|  | Adjusted R^2^ = 0.09 | Adjusted R^2^ = 0.15 | Adjusted R^2^ = 0.14 |
| AQ subscale: Imagination | **0.90 (0.05 to 1.75)** | 0.97 (–0.05 to 1.98) | 0.92 (−0.14 to 1.98) |
|  | Adjusted R^2^ = 0.04 | Adjusted R^2^ = 0.13 | Adjusted R^2^ = 0.12 |
| Note. Y-BOCS=Yale-Brown obsessive compulsive scale, AQ=Autism-spectrum quotient, FAS-SR=Family accommodation scale for OCD-self rated version. | | | |
| ^a^ Model 1 added sex, age, marital status, living with someone, household income, educational level, job status, time between appearance of symptoms and visiting hospital, FAS-PR score, and recruiting method into crude model. | | | |
| ^b^ Model 2 added well-being score into Model 1. | |  |  |

| Supplementary Table 3 Association between AQ Total score and each sub-scale score and well-being using internet-survey participants. | | | |
| --- | --- | --- | --- |
| Predictors | Crude model | Model 1^a^ | Model 2^b^ |
|  | *β* (95%CI) | *β* (95%CI) | *β* (95%CI) |
| AQ Total score | **–0.28 (–0.47 to –0.10)** | −0.21 (−0.44 to 0.01) | −0.20 (−0.44 to 0.03) |
|  | Adjusted R^2^ = 0.08 | Adjusted R^2^ = 0.04 | Adjusted R^2^ = 0.03 |
| AQ subscale: Social skill | **–0.77 (–1.27 to –0.27)** | **−0.68 (−1.26 to −0.11)** | **−0.66 (−1.25 to −0.07)** |
|  | Adjusted R^2^ = 0.09 | Adjusted R^2^ = 0.07 | Adjusted R^2^ = 0.06 |
| AQ subscale: Attention switching | –0.49 (–1.1 to 0.20) | –0.09 (–0.90 to 0.71) | –0.04 (–0.86 to 0.77) |
|  | Adjusted R^2^ = 0.01 | Adjusted R^2^ = –0.01 | Adjusted R^2^ = –0.01 |
| AQ subscale: Attention to detail | 0.04 (–0.57 to 0.66) | 0.11 (−0.56 to 0.78) | 0.13 (−0.55 to 0.81) |
|  | Adjusted R^2^ = −0.01 | Adjusted R^2^ = –0.01 | Adjusted R^2^ = –0.01 |
| AQ subscale: Communication | –0.45 (–0.95 to 0.05) | −0.30 (−0.87 to 0.27) | −0.26 (−0.85 to 0.33) |
|  | Adjusted R^2^ = 0.02 | Adjusted R^2^ = 0.01 | Adjusted R^2^ = –0.001 |
| AQ subscale: Imagination | **–0.73 (–1.33 to –0.13)** | **–0.82 (–1.58 to –0.07)** | **–0.79 (–1.57 to –0.01)** |
|  | Adjusted R^2^ = 0.05 | Adjusted R^2^ = 0.06 | Adjusted R^2^ = 0.05 |
| Note. Y-BOCS=Yale-Brown obsessive compulsive scale, AQ=Autism-spectrum quotient, FAS-SR=Family accommodation scale for OCD-self rated version. | | | |
| ^a^ Model 1 added sex, age, marital status, living with someone, household income, educational level, job status, time between appearance of symptoms and visiting hospital, FAS-PR score, and recruiting method into crude model. | | | |
| ^b^ Model 2 added Y-BOCS score into Model 1. | |  |  |
