# Supplementary figures and images for: Associations of Autism Traits With Obsessive Compulsive Symptoms and Well-Being in Patients With Obsessive Compulsive Disorder: A Cross-Sectional Study
Source: Front Psychol. 2021 Jul 30;12:697717. doi: 10.3389/fpsyg.2021.697717 (PMC8360877; doi:10.3389/fpsyg.2021.697717)

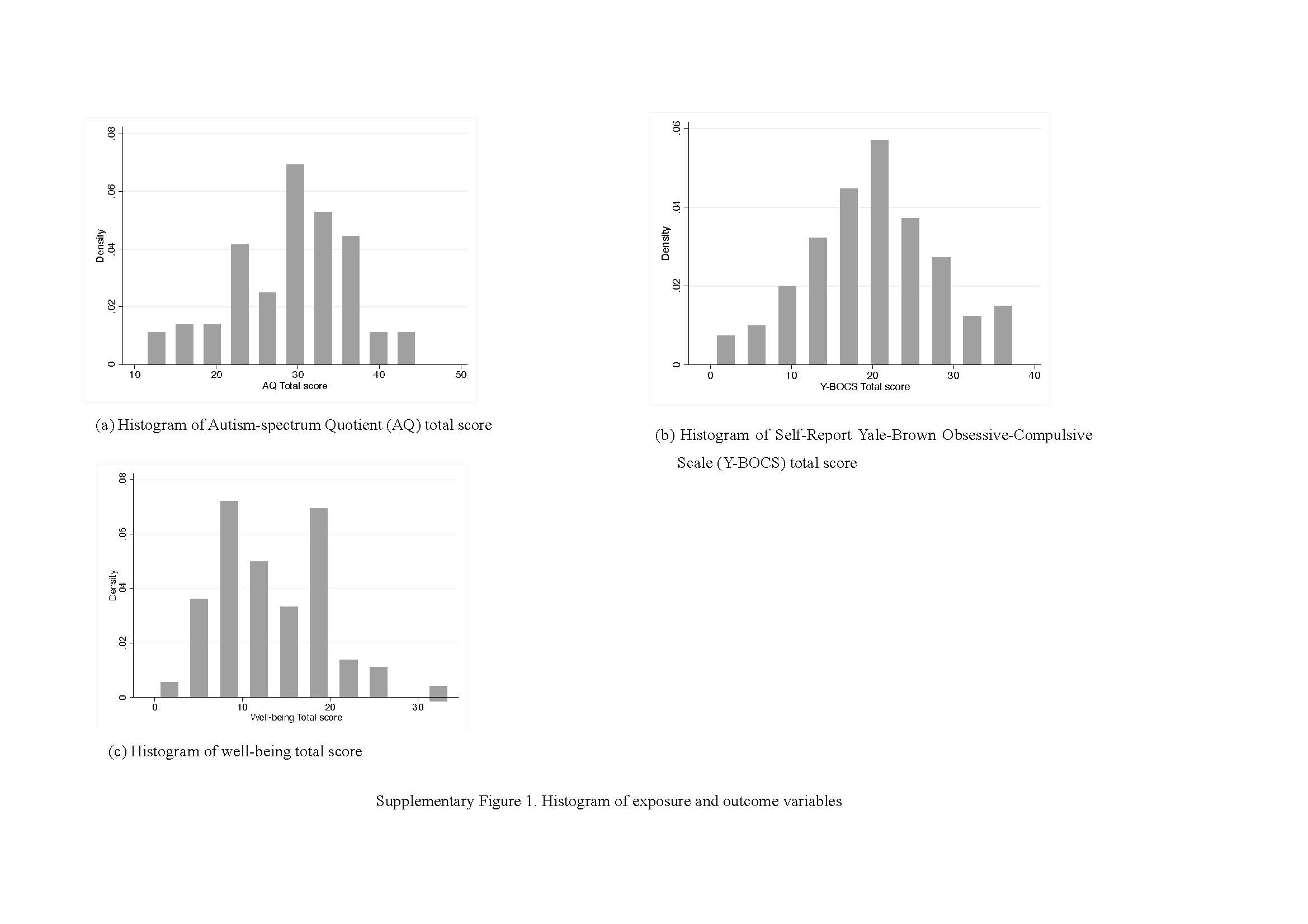

Supplement: Supplementary file 2 [file Image_1.JPEG]
